# Extensive diversification is a common feature of *Pseudomonas aeruginosa* populations during respiratory infections in cystic fibrosis^☆^

**DOI:** 10.1016/j.jcf.2013.04.003

**Published:** 2013-12

**Authors:** Abdul Ashish, Steve Paterson, Eilidh Mowat, Joanne L. Fothergill, Martin J. Walshaw, Craig Winstanley

**Affiliations:** a Regional Adult Cystic Fibrosis Unit, Liverpool Heart and Chest Hospital, Liverpool L14 3PE, United Kingdom; b Institute of Integrative Biology, University of Liverpool, Liverpool, United Kingdom; c Institute of Infection & Global Health, University of Liverpool, Liverpool, United Kingdom

**Keywords:** *Pseudomonas aeruginosa*, Cystic fibrosis, Population biology

## Abstract

**Background:**

Populations of the Liverpool Epidemic Strain (LES) of *Pseudomonas aeruginosa* undergo extensive diversification in the cystic fibrosis (CF) lung during long-term chronic infections.

**Methods:**

We analyzed sets of 40 isolates from the sputa of five CF patients, each chronically infected with a different non-LES strain of *P. aeruginosa*. For each sample (two per patient), diversity was assessed by characterizing nine phenotypic traits.

**Results:**

All *P. aeruginosa* populations were highly diverse, with the majority of phenotypic variation being due to within-sample diversity.

**Conclusions:**

Maintenance of diverse populations in the CF lung is a common feature of *P. aeruginosa* infections.

## 1 Introduction

During chronic lung infections of cystic fibrosis (CF) patients *Pseudomonas aeruginosa* adapts by accumulating mutations associated with phenotypic adaptations, leading to populations of *P. aeruginosa* composed of multiple clones with differing antimicrobial susceptibility profiles [1,2]. Previously [3], we analyzed *P. aeruginosa* sputum populations from ten CF patients each infected with an important transmissible strain, the Liverpool Epidemic Strain (LES) [4,5], and showed that LES populations were highly diverse and dynamic during CF infections [3].

Studies using sequential isolates have demonstrated the accumulation of particular mutations that indicate adaptation to the CF lung [6–9]. A recent study of strain DK2, infecting multiple patients in Denmark, suggested that after the accumulation of mutations during the early stages of infection, a homogeneous population of DK2 emerged [10], appearing to contradict our observations.

Here, we examined the diversity of *P. aeruginosa* populations in five adult CF patients each chronically infected with a different non-LES strain of *P. aeruginosa*. To test whether extensive diversification is a feature unique to the LES, or common to *P. aeruginosa* infections of CF patients in general, we compared LES and non-LES populations from matched chronically infected adult CF patients.

## 2 Materials and methods

### 2.1 Patients and samples

Sputum samples were collected for routine diagnostic purposes from adult CF patients (CF20–CF24; Table 1) chronically infected (> 5 years) with different non-LES strains of *P. aeruginosa* in 2009–2010. Strains were genotyped using an ArrayTube system [11] and identified according to the hexadecimal code generated by this method as genotypes 2F82 (CF21), 2C1A (CF22; Midlands 1 strain [12]), 0F1A (CF23), C80A (CF24) and AF9A (CF25). Samples taken during periods of exacerbation (defined as previously [13]) were sub-divided into two categories: beginning of an exacerbation (acute 1), before intravenous antibiotic treatment had commenced; typically 3–7 days after admission (acute 2). For each patient included in this study, one acute 1 and one acute 2 sample was used. The five patients chronically infected with non-LES strains were matched with five LES-infected patients (Table 1). Comparison data for LES-infected patients was taken from a previous study [3]. As far as possible, patients with similar age, lung function (FEV_1_) and BMI were selected. This study was approved by the Local Research Ethics Committee (REC reference 08/H1006/47).

### 2.2 Microbiology and phenotypic/genotypic tests

*P. aeruginosa* was isolated from sputum samples as described previously [2]. From each sputum sample, 40 single colonies were analyzed, ensuring that each different colony morphology type was represented in proportion to their abundance. Isolates, confirmed as *P. aeruginosa* but non-LES using PCR assays [12,14], were analyzed for nine phenotypic traits: colony morphology, auxotrophy, hypermutability [15], and susceptibility to six antimicrobial agents (tobramycin, colistin, ceftazidime, ciprofloxacin, meropenem and tazobactam/pipericillin) [3].

### 2.3 Statistical analyses

In order to estimate the population differentiation between patients, between samples within patients and between isolates within samples, we performed hierarchical analysis of variance using the ade4 package in R (r-project.org). We define a sub-type as a specific combination of trait values, and the phenotypic distance between a pair of sub-types as the number of traits that differed between them. Sub-type diversity was calculated as the probability of two randomly picked isolates being the same sub-type based on the sub-type frequencies within a sample. Sub-type sharing between a pair of samples was calculated as the probability of a randomly picked isolate from each sample sharing the same sub-type. This sub-type sharing probability was normalised using a logit transform (log[p / (1 − p)]).

## 3 Results

### 3.1 Overall *P. aeruginosa* phenotypic diversity

**Supplementary Fig. 1:**
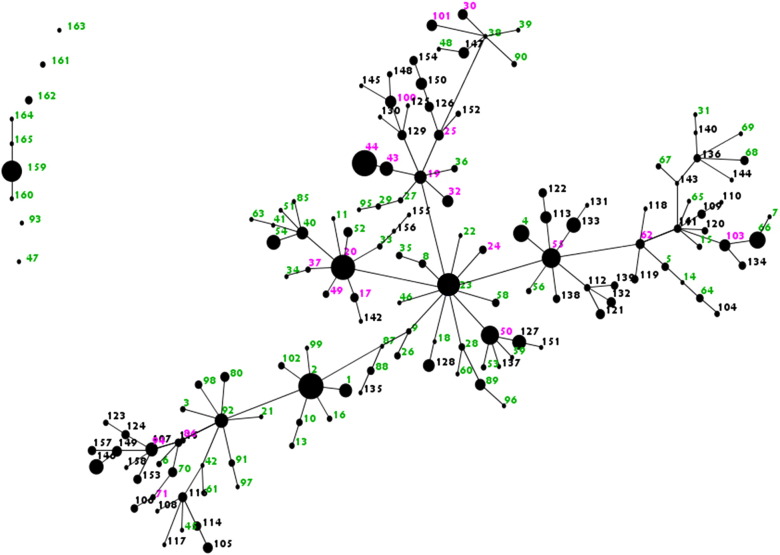
*P. aeruginosa* sub-types isolated from LES and non-LES patients. The figure shows an eBURST representation (http://eburst.mlst.net/) of all sub-types found amongst *P. aeruginosa* isolates from the five LES-infected and 5 non-LES infected CF patients, based on the nine phenotypic traits tested. Each dot represents a different sub-type. Sub-types differing in one characteristic are linked by a single band. The size of a dot reflects the relative abundance of the sub-type. The sub-types are arbitrarily numbered. Pink numbers represent sub-types found in both groups (LES and non-LES); Green numbers and black numbers represent sub-types found only in the LES group and non-LES group respectively.



High phenotypic diversity was apparent in the *P. aeruginosa* populations from each of the chronically infected CF patients. Based on the nine phenotypic traits analyzed, 400 isolates taken from 10 sputum samples from non-LES-infected patients comprised a total of 75 distinct phenotypic sub-types. The number of sub-types present within each patient is shown in Table 1, along with the frequency of each phenotypic characteristics measured. When the data from LES-infected and non-LES-infected patients were analyzed together, there were 152 sub-types of *P. aeruginosa* present in total, of which 97 were found only in LES-infected patients, 76 were found only in non-LES-infected patients, and 21were shared between the two groups (see Supplementary Fig. 1). There was a mean of 10 sub-types per set of 40 isolates for samples from non-LES-infected patients, compared to 10 for samples from LES-infected patients.

### 3.2 Sub-type variation in individual patients

**Fig. 1:**
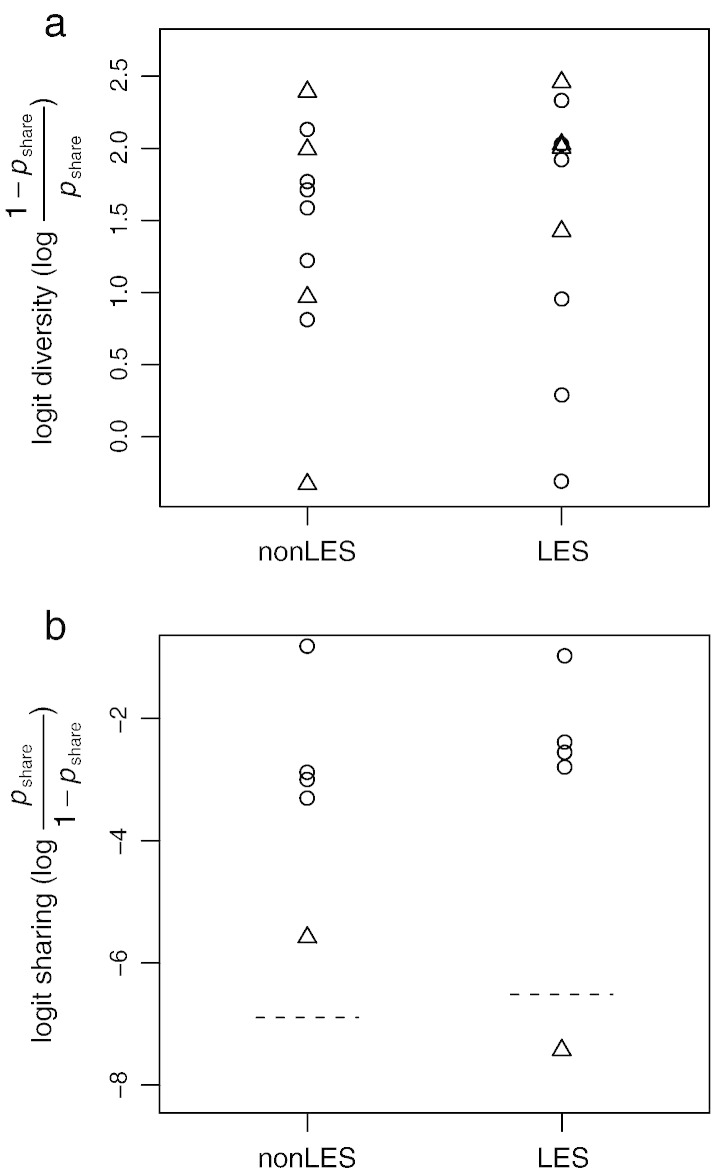
Comparisons between non-LES and LES samples. (a) Diversity within LES and non-LES samples is calculated from the probability of two isolates within a sample being of the same sub-type (*p*_share_). Circles and triangles represent samples from the beginning and end of an exacerbation, respectively. No statistically differences in diversity were observed, either between non-LES and LES samples or between samples from the beginning versus end of an exacerbation. (b) Similarity between pairs of samples taken from the same patient. Similarity is calculated from the probability of two isolates from different samples being of the same haplotype (*p*_share_). Circles represent pairs of samples taken at the beginning and end of an exacerbation and, hence, less than one month apart. Triangles represent pairs of samples taken at the beginning of separate exacerbations more than two months apart. Dotted lines indicate the mean level of similarity between samples from different patients within either the non-LES or the LES groups.

Hierarchical analysis of variance was performed on LES and non-LES groups separately to estimate the proportion of phenotypic variation attributable to (i) variation among patients, (ii) variation among samples within patients, and (iii) variation among isolates within samples. In both groups, the greatest contribution to overall diversity was due to phenotypic diversity between isolates within samples (LES-infected patients, 83%; non-LES infected patients, 81%). Overall, the LES and non-LES infections exhibited equivalent levels of diversity within a single sputum sample (Fig. 1a) and an equivalent degree of correlation between different sputum samples taken from the same patient (Fig. 1b).

### 3.3 Frequency of phenotypic traits

There were differences between the groups (non-LES-infected versus LES-infected patients) with respect to some of the phenotypes tested. For example, there was a far greater range in prevalence of isolates exhibiting the hypermutability phenotype amongst the non-LES samples [Non-LES (0–61%), LES (2–12%)]. Susceptibilities to commonly used antibiotics varied considerably within patient samples, with the exception of colistin (Table 1).

## 4 Discussion

Previous studies, tending to focus on small numbers of sequential isolates [6–8], have concluded that *P. aeruginosa* adapts in specific ways, including loss of virulence. Yet, it has been demonstrated that concurrent pairs of isolates can also vary considerably from each other [16], and we have shown previously that some strains exhibit dynamic turnover of sub-types exhibiting different phenotypic and genotypic characteristics [3]. It has been suggested that some strains reach an evolutionary plateau, characterized by low phenotypic variations [10], leading us to wonder whether the extensive diversification seen with chronic LES infections may be a particular feature of this strain. Here we analyzed samples from multiple adult CF patients infected with non-LES *P. aeruginosa* strains and compared them with equivalent samples from LES-infected patients. Our observations suggest that the extensive diversity reported previously is a widespread feature of *P. aeruginosa* populations in the lungs of CF patients who have been chronically infected for long periods. For both groups (LES and non-LES), the greatest contribution to the diversity in sub-types observed was within individual sputum samples, rather than because of variation between patients, even though the non-LES group patients were each infected with a different strain.

The wider range of frequencies of some of the phenotypic traits measured between the LES-infected and the non-LES-infected groups is likely to be due to strain-specific variations in the non-LES group. Hence, although the extent of diversity was not different between the two groups, the actual phenotypes contributing to the variations differed. It should be noted that the isolates were not randomly selected. However, the selection criteria were the same for each sample.

This study provides further evidence that the CF lung, which constitutes a spatially heterogeneous environment with multiple discrete ecological niches, is able to sustain multiple divergent sub-types of *P. aeruginosa* simultaneously, and that this is a common feature of these kinds of infections.

The following are the supplementary data related to this article.

## Figures and Tables

**Table 1:** Patient details and phenotypic characteristic exhibited by sputum isolates from patients chronically infected with *P. aeruginosa*.

Patient	Patient characteristics				Total	nSub^a^	Mutations (%)		Antibiotic resistance (%)						Colony morphology type (%)					
	Sex	Age (y)	FEV_1_ (%)	BMI	n		HM	Aux	TOB	COL	CEF	CIP	MER	TAZ	GM	GNMS	Mtr	MWO	StNMS	RM
*Non-LES*																				
CF20	M	26	41	23	80	22	25	95	61	0	75	42	20	17.5	2	48	0	2	48	0
CF21	M	30	43	15	80	15	61	10	0	0	25	50	20	0	0	47	0	3	50	0
CF22	M	21	38	16	80	8	0	100	0	0	2	5	0	0	2	76	0	0	22	0
CF23	M	21	38	21	80	23	0	71	2	0	31	21	34	24	0	0	0	25	75	0
CF24	M	22	54	20	80	16	22	0	0	0	61	90	46	0	0	76	0	24	0	0
All (mean)		24	43	19	400	16.8	22	55	13	0	39	42	24	8	1	49	0	11	39	0

*LES*																				
CF3	F	22	66	28	80	19	2	38	61	0	70	4	60	2	42	0	2	0	56	0
CF4	M	33	45	19	80	31	8	12	12	0	10	16	2	2	4	36	0	34	24	2
CF7	F	24	40	17	80	19	2	18	35	0	35	14	0	0	0	0	8	52	42	0
CF8	F	27	39	18	80	16	5	52	8	0	55	12	94	48	72	14	0	12	0	2
CF10	F	24	40	17	80	25	12	4	82	0	66	8	66	18	0	48	0	4	48	0
All (mean)		26	46	20	400	21.6	6	25	40	0	47	11	44	14	24	20	2	20	34	1

Abbreviations: FEV_1_, forced expiratory volume in 1 s; BMI, body mass index; HM, hypermutable phenotype; Aux, auxotrophy; TOB, tobramycin; COL, colistin; CEF, ceftazidime; CIP, ciprofloxacin; MER, meropenem; TAZ, tazobactam/pipericillin; GM, green mucoid; GNMS, green non-mucoid smooth; Mtr, mucoid transparent; MWO, mucoid white opaque; RM, red mucoid; StNMS, straw coloured non-mucoid smooth.

a nSub indicates the total number of different sub-types for each set of 80 isolates.

## References

[1] Foweraker J.E., Laughton C.R., Brown D.F., Bilton D. (2005). Phenotypic variability of *Pseudomonas aeruginosa* in sputa from patients with acute infective exacerbation of cystic fibrosis and its impact on the validity of antimicrobial susceptibility testing. J Antimicrob Chemother.

[2] Fothergill J.L., Mowat E., Ledson M.J., Walshaw M.J., Winstanley C. (2010). Fluctuations in phenotypes and genotypes within populations of *Pseudomonas aeruginosa* in the cystic fibrosis lung during pulmonary exacerbations. J Med Microbiol.

[3] Mowat E., Paterson S., Fothergill J.L., Wright E.A., Ledson M.J, Walshaw M.J. (2011). *Pseudomonas aeruginosa* population diversity and turnover in cystic fibrosis chronic infections. Am J Respir Crit Care Med.

[4] Winstanley C., Langille M.G.I., Fothergill J.L., Kukavica-Ibrulj I., Paradis-Bleau C., Sanschagrin F. (2009). Newly introduced genomic prophage islands are critical determinants of in vivo competitiveness in the Liverpool Epidemic Strain of *Pseudomonas aeruginosa*. Genome Res.

[5] Fothergill J.L., Walshaw M.J., Winstanley C. (2012). Transmissible strains of *Pseudomonas aeruginosa* in cystic fibrosis lung infections. Eur Respir J.

[6] Smith E.E., Buckley D.G., Wu Z., Saenphimmachak C., Hoffman L.R., D'Argenio D.A. (2006). Genetic adaptation by *Pseudomonas aeruginosa* to the airways of cystic fibrosis patients. Proc Natl Acad Sci U S A.

[7] Bragonzi A., Paroni M., Nonis A., Cramer N., Montanari S., Rejman J. (2009). *Pseudomonas aeruginosa* microevolution during cystic fibrosis lung infection establishes clones with adapted virulence. Am J Respir Crit Care Med.

[8] Huse H.K., Kwon T., Zlosnik J.E., Speert D.P., Marcotte E.M., Whiteley M. (2010). Parallel evolution in *Pseudomonas aeruginosa* over 39,000 generations in vivo. mBio.

[9] Cramer N., Klockgether J., Wrasman K., Schmidt M., Davenport C.F., Tummler B. (2011). Microevolution of the major common *Pseudomonas aeruginosa* clones C and PA14 in cystic fibrosis lungs. Environ Microbiol.

[10] Yang L., Jelsbak L., Marvig R.L., Damkiaer S., Workman C.T., Rau M.H. (2011). Evolutionary dynamics of bacteria in a human host environment. Proc Natl Acad Sci U S A.

[11] Wiehlmann L., Wagner G., Cramer N., Siebert B., Gudowius P., Morales G. (2007). Population structure of *Pseudomonas aeruginosa*. Proc Natl Acad Sci U S A.

[12] Fothergill J.L., Upton A.L., Pitt T.L., Hart C.A., Winstanley C. (2008). Diagnostic multiplex PCR assay for the identification of the Liverpool, Midlands 1 and Manchester CF epidemic strains of *Pseudomonas aeruginosa*. J Cyst Fibros.

[13] Goss C.H., Burns J.L. (2007). Exacerbations in cystic fibrosis. 1: epidemiology and pathogenesis. Thorax.

[14] De Vos D., Lim A., Pirnay J.P., Struelens M., Vandenvelde C., Duinslaeger L. (1997). Direct detection and identification of *Pseudomonas aeruginosa* in clinical samples such as skin biopsy specimens and expectorations by multiplex PCR based on two outer membrane lipoprotein genes, oprI and oprL. J Clin Microbiol.

[15] Kenna D.T., Doherty C.J., Foweraker J., Macaskill L., Barcus V.A., Govan J.R.W. (2007). Hypermutability in environmental *Pseudomonas aeruginosa* and in populations causing pulmonary infection in individuals with cystic fibrosis. Microbiology.

[16] Chung J.C., Becq J., Fraser L., Schulz-Trieglaff O., Bond N.J., Foweraker J. (2012). Genomic variation among contemporary *Pseudomonas aeruginosa* isolates from chronically infected cystic fibrosis patients. J Bacteriol.

