# Supplementary material for: Extensive diversification is a common feature of Pseudomonas aeruginosa populations during respiratory infections in cystic fibrosis
Source: J Cyst Fibros. 2013 Dec;12(6):790–3. doi: 10.1016/j.jcf.2013.04.003 (PMC3851688; doi:10.1016/j.jcf.2013.04.003)
Supplement: Supplementary file 1 [file mmc1.pptx]

## Slide 1
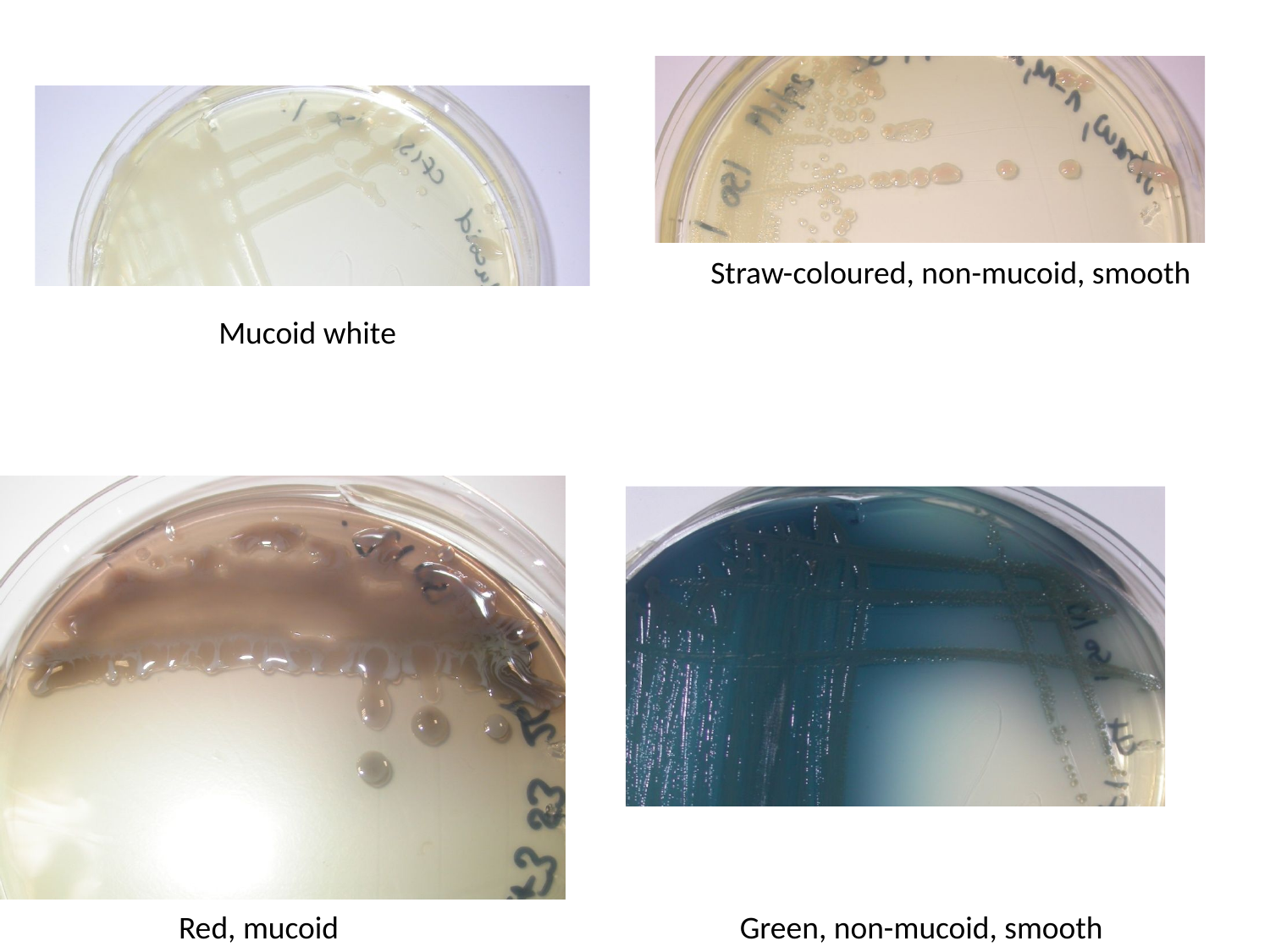

Straw-coloured, non-mucoid, smooth
Mucoid white
Red, mucoid
Green, non-mucoid, smooth

## Slide 2
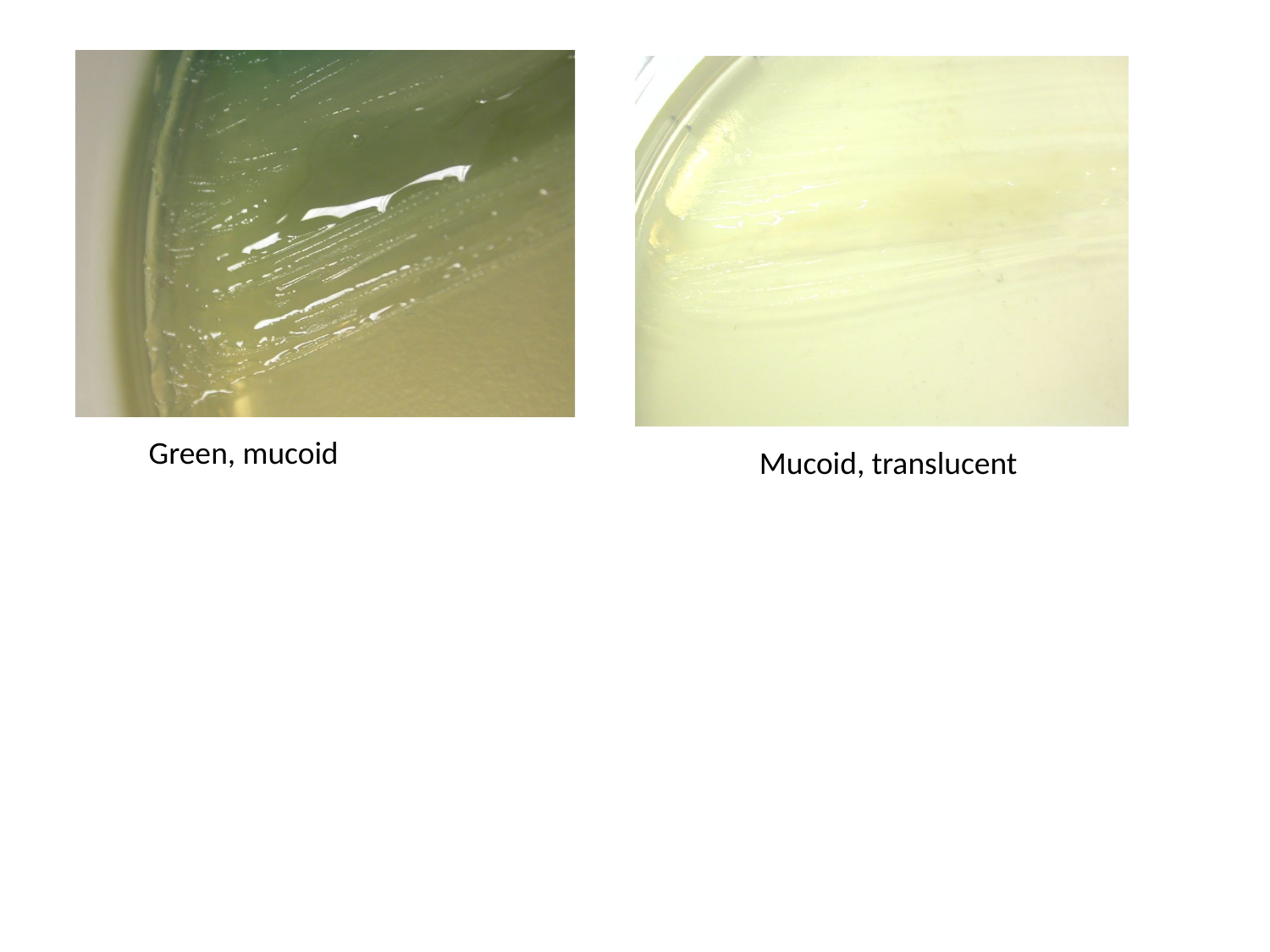

Green, mucoid
Mucoid, translucent
